# Formation of Sulfonyl Aromatic Alcohols by Electrolysis of a Bisazo Reactive Dye

**DOI:** 10.3390/molecules171214377

**Published:** 2012-12-05

**Authors:** María P. Elizalde-González, Uriel Arroyo-Abad, Esmeralda García-Díaz, Enric Brillas, Ignasi Sirés, Martín M. Dávila-Jiménez

**Affiliations:** 1 Centro de Química, Instituto de Ciencias, Universidad Autónoma de Puebla, CU, San Claudio s/n, Edif 103H, 72570 Puebla, Pue., Mexico; 2 Facultad de Ciencias Químicas, Universidad Autónoma de Puebla, CU, San Claudio s/n, Edif 105H, 72570 Puebla, Pue., Mexico; 3 Laboratori d’Electroquímica dels Materials i del Medi Ambient, Departament de Química Física, Facultat de Química, Universitat de Barcelona, Martí i Franquès 1-11, 08028 Barcelona, Spain

**Keywords:** sulfonyl aromatic alcohols, 4-(2-methoxyethylsulfonyl)benzenamine, reactive black 5, electrolysis products, logP

## Abstract

Five sulfonyl aromatic alcohols, namely 4-((2-hydroxyethyl)sulfonyl)phenol, 4-((2-(2-((4-hydroxyphenyl)sulfonyl)ethoxy)vinyl)sulfonyl)phenol, 4-(ethylsulfonyl)phenol, 4-(vinylsulfonyl)phenol and 5-((4-aminophenyl)sulfonyl)-2-penten-1-ol were identified by LC-ESI-Qq-TOF-MS as products formed by electrolysis of the bisazo reactive dye Reactive Black 5 (RB5). Since electrolyses were performed in an undivided cell equipped with Ni electrodes in alkaline medium, amines like 4-(2-methoxyethylsulfonyl)benzene-amine (MEBA) with *m/z* 216 were also suspected to be formed due to the plausible chemical reaction in the bulk or the cathodic reduction of RB5 and its oxidation by-products. Aiming to check this hypothesis, a method was used for the preparation of MEBA with 98% purity, via chemical reduction also of the dye RB5. The logP of the synthesized sulfonyl aromatic compounds was calculated and their logk_w_ values were determined chromatographically. These data were discussed in regard to the relationship between hydrophobicity/lipophilicity and toxicity.

## 1. Introduction

Electrolysis can be considered as a physicochemical method for both the decomposition of environmental pollutants (electrodegradation) and the production of new chemical compounds (electrosynthesis). According to this point of view, a target pollutant functioning as remediation object, may serve also as reagent in a synthesis of new products. The well-known disazo dye Reactive Black 5 (RB5) has usually been chosen as a model organic pollutant to assess the performance of biodegradation [1,2,3,4], photoelectrochemical degradation [5,6] and electrodegradation [7,8,9,10] technologies. Electro-oxidation with anodes like Pt, platinised Ti, BDD, Ni, RuO_2_ and IrO_2_ has been the most applied electrochemical technology for the decolorization and mineralization of such dye solutions [5,6,7]. According to several recent comprehensive reviews, these electrode materials allow the production of large amounts of ^·^OH by simply controlling the applied current [11,12,13] and can lead to the formation of many different compounds. The identification of the reaction products has been most typically performed by means of chromatographic techniques coupled to mass spectrometry [2,3,4,6,7,8]. However, the identification of the products generated upon the application of a remediation treatment to polluted reservoirs or solutions tends to be certainly troublesome because in many cases there are no available standard compounds to confirm the hypothesized products [14,15]. In addition, the toxicity of the treated solutions has been largely demonstrated [5], which can be accounted for by the accumulation of hazardous by-products arising from some of the decomposition routes. In our former studies on RB5 [16,17], no products were identified. However, more recently, we have reported some of the products formed in the cathodic and anodic chambers of a divided cell [18].

A vast number of by-products can be formed during the treatment of waters containing an organic pollutant. For example, in the case of bioremediation, it has been reported that the anaerobic treatment of hydrolyzed RB5 yields 39% of unknown products [2]. These products cannot be identified, mainly due to the absence of spectra libraries for sulfonated compounds chromatographed with coupling to electrospray ionization MS. Previous studies on the identification of the products formed during the electrolysis of RB5 have applied LC coupled to MS and MS/MS detectors. But, the use of the Qq-TOF-MS detector becomes much more interesting, since the exact masses of oxidation products can be ascertained. This advantage allows a much more reliable, unequivocal identification in comparison to that provided by the simple nominal masses found in other works [2,3,4,7,8].

The first purpose of the present paper was to employ the electrolysis of a model reactive bisazo dye (RB5) solution as a parallel synthetic procedure to form products not previously reported. The second task was to perform their structural elucidation using several techniques, namely LC-DAD, LC-ESI-TOF-MS and LC-ESI-Qq-TOF-MS.

The presence of Cl^−^ and SO_4_^2−^ ions leads to the formation of oxidizing agents like Cl_2_ and S_2_O_8_^2−^ which reroute the reaction by indirect pathways. Schellenträger demonstrated for the dye RB5 for example, that the oxidation product reported by Pham [4] with the *m/z* 200 was oxidized to the product with the *m/z* 216 by attachment of a hydroxyl group when peroxodisulfate was used [19]. This author assigned to this *m/z* of 216 the formula C_8_H_11_N0_4_S corresponding to 2-amino-5-(hydroxyl-ethanesulfonyl)-phenol (AHEP). In our case, AHEP could also be formed during the electrochemical oxidation of RB5 because the reagent RB5 contains Cl^−^ (5%) and SO_4_^2−^ (28%). We tested this point previously and could establish that the basic medium was unsuitable for the formation of S_2_O_8_^2−^ and the amount of chloride present in the RB5 reagent was not sufficient for the formation of Cl_2_ during the electrochemical oxidation of the dye RB5 in an undivided cell. Nevertheless, a different plausible intermediate such as 4-(2-methoxyethylsulfonyl)benzenamine (MEBA), also with *m/z* 216, may form via reduction of the azo bond and methylation. We aimed to test the formation of MEBA potentiostatically in an undivided cell using RB5 and nickel electrodes, but MEBA is not commercially available as a reference compound. For this reason, the dye RB5 itself was used as reagent to prepare MEBA by a simple, novel chemical reduction methodology. We focused specially on its purity and aiming at working under gentle conditions. MEBA was exhaustively characterized and compared with the unidentified electrolysis products. Finally, the study also converged towards an exploration of the toxicity of the products, based on the assessment of their hydrophobicity.

## 2. Results and Discussion

Advanced oxidation processes such as heterogeneous photocatalysis and biodegradation have been used plentifully to study the decolorization of RB5 solutions. In contrast, the implementation of electrochemical processes for the treatment of water containing RB5 is less documented [5,7,8,9,10,20,21,22]. Furthermore, only some few reports have dealt with the identification of the RB5 electrolysis products [7,8,18], which is mainly due to the lack of proper analytical equipment and standard reagents.

### 2.1. Identification of Products Formed by Electrolysis

**Figure 1:**
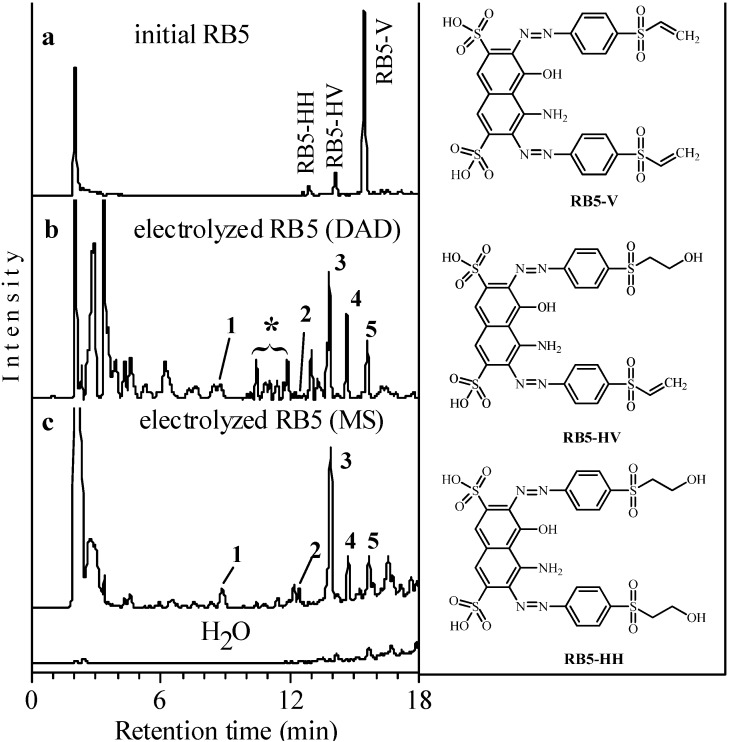
(**a**) TIC obtained for the LC-MS analysis of the initial solution of the reagent RB5. (**b**) LC-DAD chromatogram with baseline correction of the RB5 solution after electrolysis. (**c**) TIC for the same solution, compared to that of water.

The total ion chromatogram (TIC) obtained for the LC-MS analysis of the initial RB5 solution is shown in Figure 1a. On the right side of this figure, the molecular structures for the three peaks detected are depicted. As can be observed, the alkaline solution mainly contains the vinyl form (RB5-V) of the dye that appears at *t*_R_ = 15.4 min with *m/z* 706.0068 (Δ*m* 2.8). Other minor identified species in the initial solution were the partially hydrolyzed vinyl form (RB5-HV) at *t*_R_ = 14.1 min, with *m/z* 724.0169 (Δ*m* 2.2) and the completely hydrolyzed form (RB5-HH) at *t*_R_ = 12.9 min, with *m/z* 742.0261 (Δ*m* 0.3).

**Figure 2:**
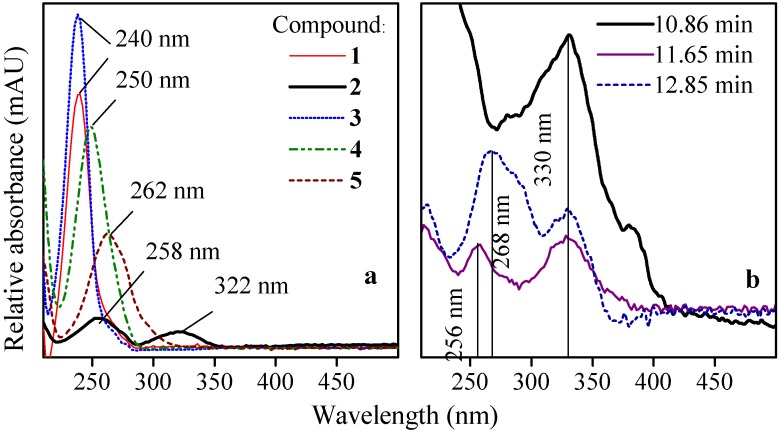
(**a**) UV/Vis spectra for: products **1**–**5** indicated in Figure 1**b**,**c** for the resulting mixture and (**b**) other peaks at different retention times.

The chromatograms obtained in the LC-DAD and LC-MS analyses of the products formed by electrolysis of the RB5 solution at a cathodic potential of −3 V *vs.* SCE (*I* = 380 mA) and with *E*_cell_ = 4.0 V for 90 min are presented in Figure 1b,c, respectively, and reveal five main products formed by electrolysis. Peaks **1**–**5** exhibited well-defined UV/Vis spectra, which informed about the similarities between the aromatic moieties, as can be seen in Figure 2a. Peak **2** with bands at 258 and 322 nm presented much less similarities among these compounds. This may suggest the existence of transitions occurring in naphthalene or conjugated biphenyl structures, as it will be clearly demonstrated below. On the other hand, Figure 2b shows that peaks appearing within 10 and 13 min, marked with an asterisk in Figure 1b, exhibited less characteristic bands in their UV spectra, although they still maintain the aromaticity.

The TIC shown in Figure 1c was obtained in negative ionization mode. It can be seen that the peaks detected by DAD and marked with an asterisk in Figure 1b could not be detected by MS. Positive-ion (ESI(+)) full scans of the electrolyzed solution, recorded over the *m/z* range 100–1,000, did not yield signals in the LC-ESI-(Qq)-TOF-MS chromatograms for any of the products. This could be attributed to their low concentration and/or ionization capability in the positive mode.

**Figure 3:**
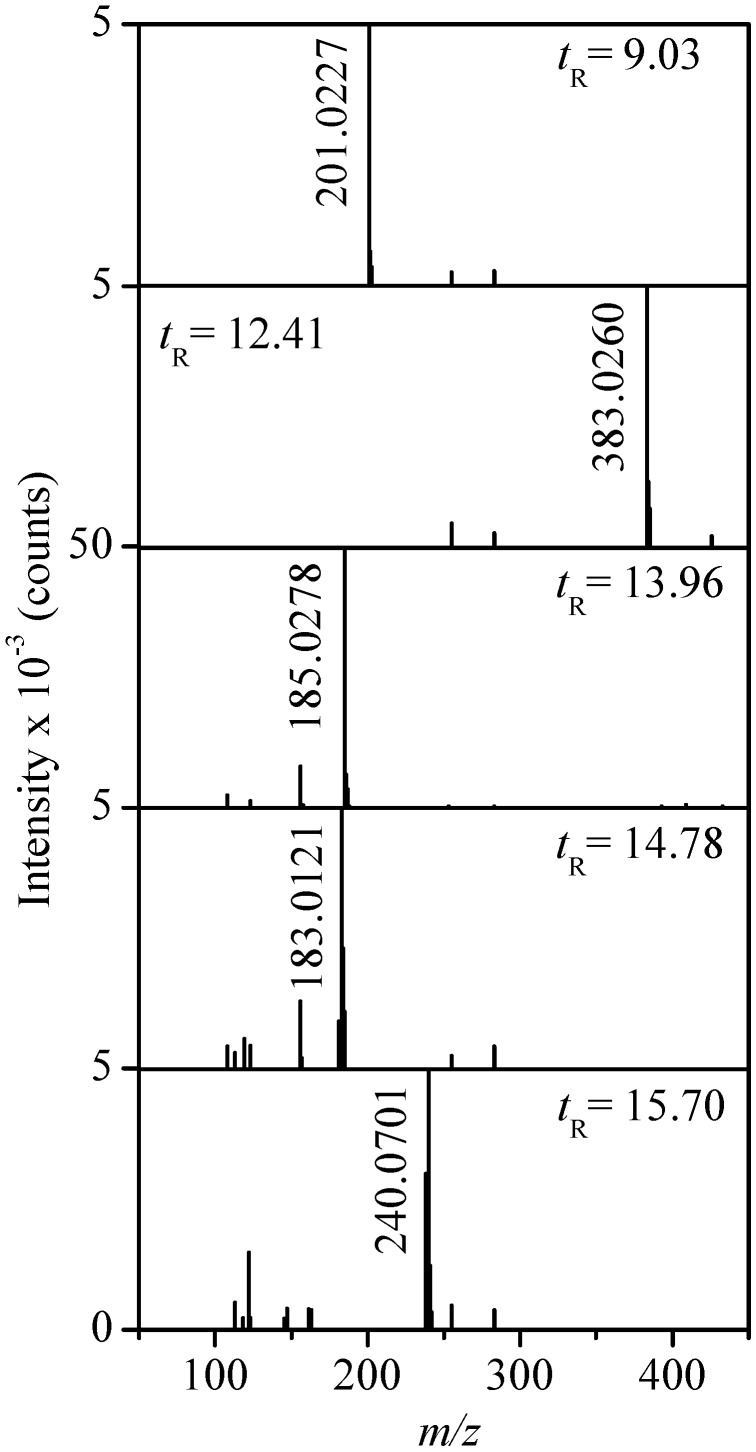
MS spectra of peaks **1**–**5** indicated in the TIC of Figure 1c.

The MS spectra of the five products included in Figure 1c acquired in ESI(−) mode, are then shown in Figure 3 and the proposed formulae have been summarized in Table 1 and Table 2, thus revealing that mainly sulfonyl aromatic alcohols are formed. The elucidation of the structure of compound **2** with two aromatic rings confirmed the presence of the two bands observed in the UV/Vis spectrum of Figure 2a.

**Table 1:** Formulae and relevant mass spectrometry data for the sulfonyl aromatic alcohols (**1**–**5**) identified by LC-ESI(-)-(Qq)-TOF-MS as electrolysis products of RB5, as well as for MEBA.

Compd.	Condensed formula	*t*_R_ (min)	[M-H]_exper._	[M-H]_calc._	Δ *m* (ppm)	Reported by
**1**	C_8_H_10_O_4_S	9.03	201.0227	201.0227	0.0	[18]
**2**	C_16_H_16_O_7_S_2_	12.41	383.0260	383.0265	−1.3	new
**3**	C_8_H_10_O_3_S	13.96	185.0278	185.0278	0.0	[18]
**4**	C_8_H_8_O_3_S	14.78	183.0121	183.0121	0.0	new
**5**	C_11_H_15_NO_3_S	15.70	240.0701	240.0700	0.4	new
			[M+H]_exper._	[M+H]_calc._		
**MEBA**	C_9_H_13_NO_3_S	12.30	216.0691	216.0689	0.9	[23]

Despite the low mass errors calculated for the proposed structures, collected in Table 1, MS/MS spectra were recorded for compounds **1**–**5** and the resulting product ions, are summarized in Table 2. In our previous work [18], we produced compounds **1** and **3** by electrolysis of an alkaline RB5 solution in the anodic chamber of a divided electrochemical cell.

**Table 2:** Product ions of the electrolysis products **2**, **4** and **5** obtained upon MS/MS analysis by means of LC-ESI-Qq-TOF-MS.

Compd.	Proposed structure	[M-H]_exper._	Product ions MS/MS
**1**	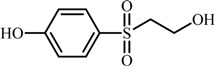	201.0227	92: [M-H-SO_2_C_2_H_5_O]^−·^
			108: [M-H-SOC_2_H_5_O]^−·^
			156: [M-H-C_2_H_5_OH]^−·^
**2**	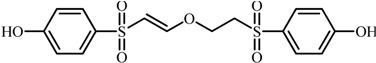	383.0260	353: [M-H-CH_2_O]^−·^
			262: [M-H-C_3_H_5_O_3_S]^−·^
			230: [M-H-C_3_H_5_O_5_S]^−·^
			199: [M-H-C_8_H_8_O_3_S]^−·^
			184: [M-H-C_8_H_7_O_4_S]^−·^
			169: [M-H-C_9_H_10_O_4_S]^−·^
**3**	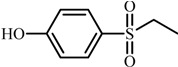	185.0278	92: [M-H-C_2_H_5_O_2_S]^−·^
			108: [M-H-C_2_H_5_OS]^−·^
			156: [M-H-C_2_H_5_]^−·^
**4**	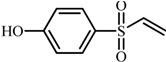	183.0121	92: [M-H-C_2_H_5_]^−·^
			108: [M-H-C_2_H_3_OS]^−·^
			156: [M-H-C_2_H_5_]^−·^
**5**	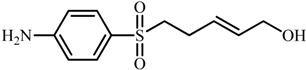	240.0701	107: [M-H-C_2_H_5_NO_2_S]^−·^
			147: [M-H-CH_3_NO_2_S]^−·^
			163: [M-H-CHO_2_S]^−·^
			184: [M-H-C_3_H_4_O]^−·^
**MEBA**	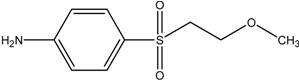	[M+H]_exper._ 216.0691	

The amino form of the vinyl compound **4** depicted in Table 2 has also been confirmed as a biodegradation product of RB5 arising from anaerobic reduction process [3,24]. The same authors also reported a di-*p*-aminobenzeneethylsulfonic ether with *m/z* 383.5 differing slightly from compound **2** (*m/z* 383.0260, Δ*m* −1.3 ppm) identified by us. The structure that we propose here for compound **2** contains an aliphatic C=C bond, as well as terminal hydroxyl groups instead of the amino groups indicated by Plum and Rehorek [3,24]. This discrepancy can be due to the strong alkaline conditions used by us or to the proper accuracy of the TOF mode or, more likely, to the singular results yielded by the reductive biodegradation compared to the electrolysis in an undivided cell, where redox processes can take place. In total anaerobic reduction, auto-oxidation reactions of *o*-aminohydroxynaphthalenes are conceived [24,25]. Similarly, the hydroxylated compounds **2** and **4** could then correspond to electro-oxidation products of the amino compounds reported by Rehorek and Plum, upon participation of strong oxidizing species such as hydroxyl radicals (^·^OH) formed at Ni anodes [18].

The collision energy for the MS/MS runs was set at a relative high value (25–30 V) in order to observe a larger number of product ions, as confirmed in Table 2. Moreover, the above mentioned amino form of compound **2** with a reported *m/z* 383.5 [3] would have an exact *m/z* 383.0741, which differs slightly from the *m/z* 383.0260 (Δ*m* −1.3 ppm) corresponding to the hydroxylated form being suggested in the present paper.

Since amino, hydroxylated and methylated derivatives of the arylsulfonyl structure were produced by electrolysis of RB5 solutions, the identity of the peaks with an asterisk in Figure 1b may correspond to methylated arylsulfonyl amines. The synthesis of aromatic amines is often difficult. Methods based on the electrophilic aromatic substitution involving nitration followed by reduction or direct amination are incompatible with many functional groups. However, the chemical reduction of RB5 can yield arylsulfonyl amines, which may serve as standards for the identification of possible electrolysis products of RB5. Moreover, a methoxy group can be introduced into the arylsulfonyl amine by completing the chemical reduction of RB5 in an appropriate medium. We put forward the synthesis of MEBA (see molecular formula in Table 2), for which two procedures were reported elsewhere: (i) the treatment of *p*-acetanilidosulfinic acid with alkoxyethyl chloride [26] and (ii) the use of an addition reaction employing aryl β-chloroethyl sulfones prepared from β-hydroxyethyl sulfides and thionyl chloride [23].

### 2.2. Consideration of MEBA as One of the Unidentified Electrolysis Products

**Figure 4:**
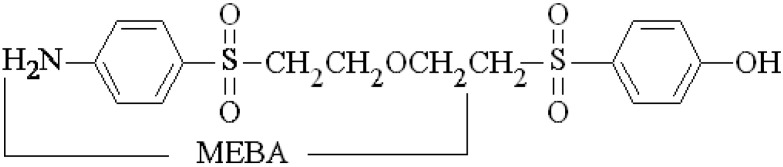
Condensation product formed during the electrolysis of RB5 in a divided cell.

Other experiments performed by us in a divided cell demonstrated that during the electrolysis of 80 mg dm^−3^ RB5 at a cathodic potential of −3 V *vs.* SCE and with *E*_cell_ = 4.0 V for 120 min, the compound with *m/z* 384.0581 (Δ*m* = 1.56) depicted in Figure 4, was formed:

Its structure could correspond to a condensation product arising from smaller sulfonyl aromatic compounds. This finding, together with Schellenträger’s compound AHEP motivated the preparation of a sulfonyl derivative with *m/z* 216 carrying a terminal methyl group, *i.e*., MEBA to be used as a standard. Further, the possible formation of MEBA as one of the electrolysis products of RB5 was surveyed. The peak corresponding to MEBA appeared at *t*_R_ = 12.30 min in the TIC for an LC-MS analysis (see Table 1). On the other hand, several well-defined peaks (marked with an asterisk in Figure 1b were observed in the LC-DAD mixture of products of the electrolyzed solution in Figure 1b at *t*_R_ = 10.44, 10.89, 11.06, 11.41, 11.72 and 11.88 min. Other peaks with much lower definition, were eluted at *t*_R_ = 12.07, 12.21, 12.42 and 12.64 min. Therefore, all these latter peaks were candidates to correspond to MEBA. In principle, the following facts could support the consideration of the synthesized MEBA as one of the peaks within 12.07 and 12.64: (i) the compound was synthesized from the chemical reduction of RB5, which is a process that may also occur under the electrochemical conditions carried out and (ii) the structure should ionize positively, then being logic its absence in the TIC when doing the LC-MS analysis recorded in negative mode (see Figure 1c). By spiking the electrolyzed solution with MEBA, no height increment of none of the peaks at *t*_R_ = 12.21, 12.42, 12.64 min was observed in the DAD chromatogram. These peaks rather became overlapped, which means that MEBA eluted at a slightly different *t*_R_. This suggests that neither AHEP, nor MEBA could be synthesized by electrolysis of the dye RB5 under the conditions tested in this work.

### 2.3. Hydrophobicity/Lipophilicity/Toxicity of the Decomposition Products

Two of the most intriguing and challenging questions within the field of environmental electrochemistry are the identification and the toxicity assessment of the products formed by electrolysis. While hydrophobicity refers to the association of non-polar species in aqueous media, lipophilicity informs about the relative affinity of the molecule for lipophilic binding sites [27]. Although both terms are routinely used indistinctly, it can be argued that lipophilicity is a property determining the biological activity of a substance, whereas hydrophobicity can be considered as the anti-driving force for the transport of the pollutant in the aqueous environment. Furthermore, hydrophobicity (defined by the logarithm of the partition coefficient, logP) has shown a dependence on non-specific toxicity, mutagenicity and carcinogenicity [28]. The logP, which is normally given as the partition coefficient of a compound between octanol and water (logk_ow_), has become fundamental for the prediction of toxicity [29].

Different computational systems yield logP values diverging markedly [27,30]. We carried out the theoretical calculation of this coefficient for the identified products by using suitable software. Isocratic chromatographic runs in the reversed phase mode were also performed for the experimental determination of logk_w_, which is the extrapolated logarithm of the chromatographic capacity factor *k* for pure water. In particular, it is known that a good correlation between logk_w_ and logP or logk_ow_ (experimentally determined by the shaking flask method) is found for closely related molecules [31].

**Figure 5:**
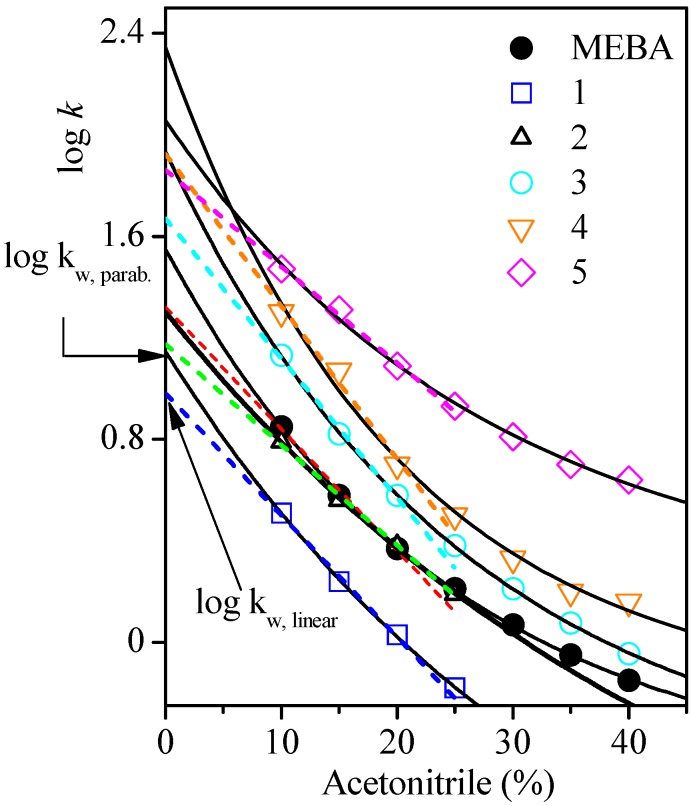
Dependence of log*k* of compounds **1**–**5** and MEBA with the content of acetonitrile in the mobile phase eluted under isocratic conditions, showing a parabolic (continuous lines) or linear (dotted lines) fitting with extrapolation up to 0% acetonitrile to yield logk_w_.

The strategy used in this part of the study consisted of three stages: (a) theoretical generation of the logk_ow_ values of the chemical structures of the compounds depicted in Table 2, (b) correlation of the calculated logk_ow_ with the logk_w_ obtained analytically from Figure 5 (extrapolation of the parabolic fitting curves—continuous lines—up to 0% acetonitrile content in the mobile phase) and (c) correlation of the calculated logk_ow_ with the logk_w_ obtained analytically at 0% acetonitrile in the same figure (extrapolation of the linear fitting curves—dotted lines—arising from data available in the range 10–25% acetonitrile). Figure 5 and Table 3 show that logk_w,parab._ > logk_w,linear_. This effect is similar to the downward curvature observed at lower methanol fractions when *n*-octanol was supplied to the mobile phase as additive for compounds with intermediate lipophilicity [32]. As demonstrated by Giaginis *et al*. [32] for a set of 45 drugs, the linear extrapolation of the logk_w_ values correlated practically 1:1 with the measured logP obtained by the shake flask method, using *n*-octanol as additive. This promoted the use logk_w,linear_ instead of logk_w,parabolic_. The logk_w,linear_ values of the products obtained by us as a result of the electrolysis of RB5 and of MEBA varied in the range 0.98–1.92 (see Table 3). For comparison, logP of aniline is 0.90, of phenol is 1.47 and, for the methyl-substituted phenols, it varies within 1.94–1.96 [29]. The data of logk_w_~logk_ow_~logP reported in the present paper for the identified products generated by electrolysis of RB5 then allow an estimation of their possible level of a toxicity, which is similar to that of phenol and its methylated derivatives.

**Table 3:** Chromatographic capacity factor *k* (= (*t*_R_ – *t*_0_)/*t*_0_, where *t*_R_ is the retention time and *t*_0_ is the dead time) under gradient conditions, experimental logk_w,parabolic_ andlogk_w,linear_, calculated logk_ow_ and total dipole moment of the identified compounds and MEBA.

Compound and IUPAC name		*k*	logk_w,parab._ (exper.)	logk_w,lin._ (exper.)	logk_ow_ (calc.)	m (Debye)
**1**	4-((2-hydroxyethyl)sulfonyl)phenol	3.4	1.14	0.98	0.37	3.8
**MEBA**	4-(2-methoxyethylsulfonyl)benzenamine	5.0	1.55	1.32	0.69	5.1
**2**	4-((2-(2-((4-hydroxyphenyl)sulfonyl)ethoxy)vinyl)sulfonyl)phenol	5.1	1.30	1.17	0.76	5.6
**3**	4-(ethylsulfonyl)phenol	5.8	1.93	1.67	1.18	5.5
**4**	4-(vinylsulfonyl)phenol	6.2	2.34	1.92	1.03	6.7
**5**	5-((4-aminophenyl)sulfonyl)-2-penten-1-ol	6.7	2.05	1.86	1.14	7.9

When the calculated logk_ow_ values were compared with the experimental logk_w,parabolic_ and the logk_w,linear_, the resulting R^2^ values were 0.8621 and 0.8848, respectively. Thus, the logk_w,linear_ data were preferably used for the examination of the elution sequence of the studied compounds, *i.e.*, to establish a congruence between their elution expressed in terms of the chromatographic retention capacity factor *k* and their experimental logk_w_ magnitudes. Usually, chromatographists search for linear dependences between log*k* and structural parameters. In the present study, such congruence can be directly related to the correct identification, that is, it serves as a complementary strategy for the verification of the proposed structures. As can be noted, despite the reduced number of data (N = 5), the chromatographic retention capacity factor *k* of the identified sulfonyl aromatic alcohols (*i.e*., excluding MEBA) formed in the RB5 solution after electrolysis, showed a sufficient linear correlation with logk_w,linear_ (R^2^ = 0.9277), with logk_ow_ (R^2^ = 0.9448) and also with the dipole moment (R^2^ = 0.9437), as can be seen in Table 4 summarizing the statistical analysis.

**Table 4:** Statistical analysis of the relationship of logk_w,linear_, logk_ow_, and the dipole moment with the elution order, expressed by the chromatographic capacity factor *k* reported in Table 3.

Statistical parameters	*k* *vs.* logk_w,linear_	*k* *vs.* logk_ow_	*k* *vs.* dipole moment
R^2^	0.9277	0.9448	0.9437
SD	0.1819	0.1273	0.5878
RSS	0.0993	0.0486	1.0364
F	18.52	24.96	24.43
P	0.0231	0.01543	0.01588

## 3. Experimental

### 3.1. General

#### 3.1.1. Materials

The dye RB5 (tetrasodium (6*Z*)-4-amino-5-*oxo*-3-[4-(2-sulfonatooxyethylsulfonyl)phenyl]diazenyl-6-[[4-(2-sulfonatooxyethylsulfonyl)phenyl] hydrazinylidene]naphthalene-2,7-disulfonate, C.I. 20505, C_26_H_21_N_5_Na_4_O_19_S_6_, CAS 12225-25-1,dye content 55%) was purchased from Sigma-Aldrich (St. Louis, MO, USA). The other required reagents were obtained from Sigma-Aldrich and Fluka (Buchs, Switzerland). Solutions of RB5 were freshly prepared in 0.1 M KOH (Merck, Darmstadt, Germany) without previous hydrolyzation and using high-purity water obtained from a Millipore Milli-Q system with resistivity >18 MΩ cm.

#### 3.1.2. Methods

The electrolysis using RB5 solutions was performed at room temperature with a conventional three-electrode system under potentiostatic conditions with a Volta Lab PGZ301 potentiostat/galvanostat using an undivided cell of 50 cm^3^ capacity. Two pieces of cylindrical Ni wire mesh with an electrode area of 40 and 70 cm^2^ were used as the cathode and anode, respectively, whereas a saturated calomel electrode (SCE) was used as the reference electrode. In all cases, the solutions were previously deaerated by bubbling pure N_2_ gas for 30 min and a flow of this gas was maintained over them during the measurements. Aqueous solutions containing 80 mg dm^−3^ RB5 and 0.1 mol dm^−3^ KOH were treated under mild electrolytic conditions in the cell by applying a cathodic potential of −3 V for 90 min. The difference between the anode and the cathode was 4.0 V. The electrolyses were performed under vigorous solution stirring with a magnetic bar to ensure a good mixing and reproducible mass transport conditions. Under these conditions, both electro-oxidation and electroreduction processes took place on the surface of the Ni anode and cathode, respectively, during the electrolysis of RB5.

#### 3.1.3. General Analytical Instrumentation

The electrolysis mixtures were analyzed by using an LC-ESI-(Qq)-TOF-MS equipment consisting of an LC Series 1260 chromatograph equipped with a degasser, binary pump and thermostated autosampler, coupled with an ESI-(Qq)-TOF-MS 6520 detector from Agilent Technologies (Santa Clara, CA, USA). For the separation, which was carried out by means of ion pair chromatography (IPC), a Macherey-Nagel Nucleodur EC C18 Isis (250 mm × 4.6 mm (i.d.), 5 µm particles) column (Düren, Germany) was used at room temperature. A mixture of 40 mmol dm^−3^ triethylammonium acetate (TEAA) as component A and acetonitrile with 0.1% HCOOH as component B was eluted at 1 cm^3^ min^−1^ as the mobile phase, with a relative percentage of A of 90% for 5 min, 10% between 5 and 25 min and 90% again up to 30 min. The injection volume was 0.1 cm^3^, whereas the dead time (*t*_0_ = 2.04 min) was determined by injecting D_2_O. The following mass spectrometry conditions were used: (i) for accurate mass analysis, ESI-TOF-MS was performed in the negative ionization mode (ESI(-)) with drying gas (N_2_) at 11 dm^3^ min^−1^, TOF fragmentor voltage of 175 V, capillary voltage of 3500 V, *m/z* range of 50–1000, gas temperature of 350 °C and nebulizer pressure of 60 psi, and (ii) for the MS/MS analyses, ESI(-)-Qq-TOF-MS was used as well applying the following particular collision energy for the identified products (in parentheses): 25 V (for **1**, **3** and **5**) and 30 V (for **2** and **4**). The abovementioned liquid chromatograph was also used to perform the LC-DAD analysis, by coupling an UV/Vis diode array detector instead. The separation conditions were analogous to those described above and the samples were monitored at *λ* = 254 nm.

#### 3.1.4. Other Techniques and Instruments Used for the Thorough Characterization of MEBA

The absorption spectrum of MEBA was recorded using a Beckman DU 7500 UV/Vis spectrophotometer. The infrared spectrum was obtained by Fourier transform infrared spectroscopy (FTIR) performed with a Nicolet Magna-750 FTIR spectrometer. Proton and ^13^C-NMR spectra were acquired with a Varian VX400 (400 MHz) spectrometer, using tetramethylsilane (TMS) as the internal standard. The differential scanning calorimetric analyses carried out for the determination of the MEBA purity were performed on a DSC Model 2010 from TA Instruments. RP-HPLC was carried out for the preparative separation of MEBA using a Beckman System Gold chromatograph equipped with an autosampler 507 and a diode-array detector selected at 210 and 265 nm, and fitted with a Macherey-Nagel Nucleosil EC 100-7-C18 [250 mm × 4.6 mm (i.d.)] column at room temperature. The mobile phase was 75:25 (v/v) methanol/water at a flow rate of 1.0 cm^3^ min^−1^.

#### 3.1.5. Calculations

The coefficients of determination R^2^, standard deviations (SD), of each linear dependence used in the work were calculated by means of the OriginPro 8.0 SR0 v8.0724 software from OriginLab Corporation (Northampton, MA, USA). The melting point of MEBA, the total dipole moment and the logarithm of the octanol-water partition coefficient (k_ow_) for the identified products were calculated by means of the Molecular Modeling Pro software from Chem SW. The most probable conformer was considered upon energy minimization and the molecular parameters were computed for the optimized geometry obtained by the Complete Neglect of Differential Overlap (CNDO) approximation.

### 3.2. Synthesis of 4-(2-Methoxyethylsulfonyl)benzenamine (MEBA)

**Scheme 1:**
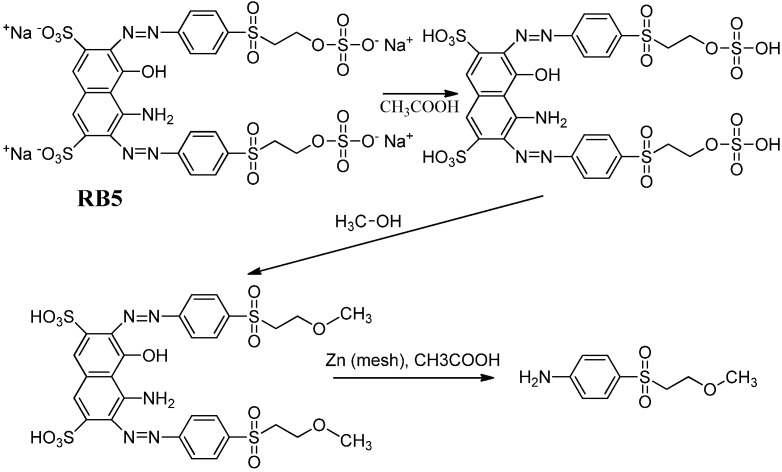
Reactions for the synthesis of MEBA.

The novel procedure for the preparation of MEBA consisted in the chemical reduction of RB5 in three consecutive steps in acetic acid/methanol medium (see Scheme 1). A mixture of RB5 (1 mmol dm^−3^), acetic acid (10 cm^3^), and Zn (7 g, 20–30 mesh) in methanol (15 mL) was stirred under a nitrogen atmosphere at 85 °C for 4.5 h and further cooled down to room temperature. Acetic acid acted first as a protonation agent, followed by methylation in the presence of methanol and final reduction with Zn to yield the amine group from the original azo bond. The mixture was then filtered, and afterwards a 1 mol dm^−3^ NaOH solution was added until the filtrate turned to alkaline. The crude of the reaction (yellow liquid) was directly extracted with diethyl ether several times and concentrated under reduced pressure on a rotary evaporator. Subsequently, it was passed through a silica gel chromatographic column with acetate/hexane as the mobile phase to collect twelve fractions. MEBA (C_9_H_13_NO_3_S), with a retention factor of *k* = 2.38, was isolated by reversed-phase (RP) HPLC after collecting the eluted fractions 8–12.

### 3.3. Characterization of MEBA

**Figure 6:**
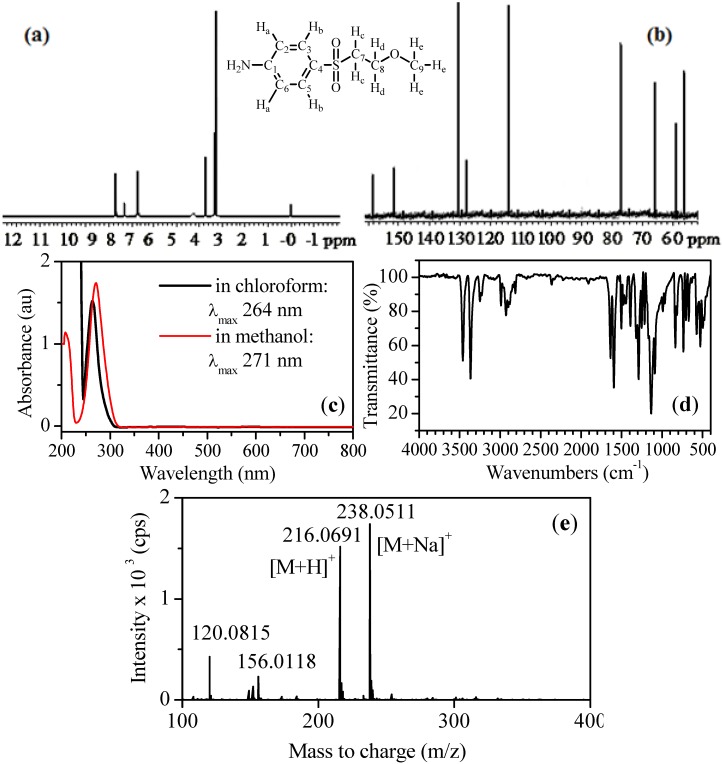
NMR (**a**,**b**), UV-Vis (**c**), FTIR (**d**) and MS (**e**) spectra of MEBA.

MEBA synthesized as explained in Section 3.2 was characterized as follows. The FTIR spectrum showed the characteristic bands of the N-H bond and C-O-C moiety of MEBA, along with those specific for the aromatic sulfonyl (-SO_2_-) substituent. The ^1^H NMR spectrum was in good agreement with the proposed structure, which was also confirmed by ^13^C-NMR spectroscopy (Figure 6a,b). A purity of 98% was confirmed by differential scanning calorimetric analysis and HPLC. The following results were obtained from the different characterization techniques: Yield = 15.04%; m.p. = 91.2 °C; UV/Vis (Figure 6c) *λ*_max_ (CH_3_OH): 271.0 nm; UV/Vis *λ*_max_ (CHCl_3_): 264.0 nm; IR *v*_max_ (KBr/cm^−1^) (Figure 6d): *str* 3459, 3365, *wag* 836 (N–H), *str* 2989, 2927 (C–H, aliphatic), *str* 2879 (H–C–O), *str* 1637, 1594 (C–C, aromatic), *str* 1288, 1137 (O=S=O), *str* 1090 (C–O–C). For the NMR (CDCl_3_) assignments, see formula of MEBA in Figure 6). ^1^H-NMR *δ* (ppm): 7.66, 7.64 (d, *J* = 8.8 Hz, 2H Hb), 6.71, 6.69 (d, *J* = 8.8 Hz, 2H Ha), 3.73, 3.71, 3.69 (t, 2H Hd), 3.35, 3.33, 3.31 (t, 2H Hc), 3.26 (s, 3H He). ^13^C-NMR *δ* (ppm): 151.47, 130.10, 127.52, 113.98, 65.90, 58.71, 56.32 (C-1, C-4, C-3,5, C-2,6, C-8, C-7, C-9). The structure was corroborated by LC-MS analysis with positive ionization (see MS spectrum in Figure 6e), where *m/z* 216.0691 was found at *t*_R_ = 12.30 min (see Table 1).

In previous synthesis of MEBA (C_9_H_13_NO_3_S) and related compounds, melting points of 194~196 °C for C_9_H_13_NO_3_S·HCl [26] and 113~115 °C for C_9_H_13_NO_3_S [23] were reported. These values differ substantially from the one obtained in the present work (91.2 °C), which is close to the one arising from our theoretical calculations (86.8 °C). Furthermore, the reported amines [23,26] were characterized uniquely by elemental analysis and, in contrast to our work, neither thermal nor chromatographic analyses were carried out to confirm their purity and structure. As a conclusion, it has been confirmed that the proposed synthesis is a good alternative to previously established routes.

## 4. Conclusions

Five sulfonyl aromatic alcohols were obtained by electrolysis of the azo dye RB5 in an undivided cell with a Ni electrode. Some of these compounds have not been previously synthesized, and the amine **5**, namely 5-((4-aminophenyl)sulfonyl)-2-penten-1-ol, has been reported for the first time. The phenolic character of two products: 4-((2-(2-((4-hydroxyphenyl)sulfonyl)ethoxy)vinyl)sulfonyl)phenol (compound **2**) and 4-(vinylsulfonyl)phenol (compound **4**) has been demonstrated and contrasted with the amino analogues reported as biodegradation products. A novel, simple method to obtain MEBA from RB5 via chemical reduction under gentle conditions has been also developed because it was a suspected electrolysis product. The synthesized MEBA was then used as standard reagent and was essential to assess the possible formation of methoxy derivatives among the electrolysis products. LC analyses and spiking trials led to conclude that MEBA can be discarded as an electrolysis product of RB5 under the current conditions. The logP values of the series of identified products obtained by electrolysis were similar to those of phenol and its methylated derivatives, therefore suggesting a similar toxicity.
